# Draft genomes of *Paenibacillus larvae* isolated from honeybee colonies (*Apis mellifera*) in Fiji

**DOI:** 10.1128/mra.01039-23

**Published:** 2023-12-22

**Authors:** Ruy Jauregui, Andrea Barcelo, Peter Bennett, Jonathan Foxwell, Oliver Quinn, Tom Rawdon, Malan Pranith Bandara, Deepika Lata, Chaminda Dissanayake, Keresi Lomata, Bede Busby, Michelle McCulley

**Affiliations:** 1 Animal Health Laboratory, Biosecurity New Zealand, Ministry for Primary Industries, St. Upper Hutt, New Zealand; 2 Animal Health Laboratory, Biosecurity Authority Fiji, Koronivia, Suva, Fiji; Indiana University, Bloomington, Indiana, USA

**Keywords:** honeybee, hive

## Abstract

Here, we report draft genomic sequences from three *Paenibacillus larvae* isolates, the causative agent of American Foulbrood (AFB), obtained from honeybee colonies of *Apis mellifera* in Fiji, which allow both enterobacterial repetitive intergenic consensus and multilocus sequence typing genotypes to be elucidated for Fijian AFB.

## ANNOUNCEMENT

*Paenibacillus larvae* was first identified in Fiji during a disease survey in 1986 (1). To date, no enterobacterial repetitive intergenic consensus (ERIC) or multilocus sequence typing (MLST) have been determined for Fijian American Foulbrood (AFB) isolates. Here, we report draft genomic sequences from three isolates, allowing both ERIC and MLST genotypes to be determined.

Samples of honey, larvae, and comb were collected from hives of *Apis mellifera* in Fiji in 2021–2022 (Table 1) and subjected to isolation and culture of *Paenibacillus larvae* following a previously described method in reference (2); briefly, samples were emulsified in 1-mL Dulbecco’s phosphate-buffered saline (DPBS) (Gibco, Life Technologies, Billings, USA), heat shocked at 80°C for 10 minutes, and inoculated on three media: non-selective Columbia sheep blood agar (BA) and selective BA with 3 µg/mL and 18 µg/mL nalidixic acid. All inoculations were incubated at 37°C, 5% CO_2_, for 48 hours. Candidate colonies were selected based on morphology and subjected to a catalase test (catalase dropper, Becton, Dickinson & Co., Franklin Lakes, USA) and then confirmed using the AFBduo real-time PCR kit (Dnature Diagnostics & Research Ltd., Gisborne, New Zealand).

**TABLE 1:** Sample sequence data and genomic statistics

Data set	Feature	Isolate		
		H2S1	BR08	Koropito
	Source	Larvae	Larvae	Honey
Nanopore	Read no.	2087505	1527207	1129772
	Coverage	381X	344X	317X
	N50/L50	560647/830	412984/836	252026/1.48 kb
	SRA link	SRR26389523	SRR26389521	SRR26389519
Illumina	Read pair no.	5539288	16625452	7600558
	Coverage	840X	2550X	1170X
	SRA link	SRR26389524	SRR26389522	SRR26389520
Assembly	Contig no.	17	23	22
	Length	4.3 Mb	4.3 Mb	4.3 Mb
	GC%	43%	43%	43%
	BUSCO score	99.2%	99.2%	99.2%
	N50	3	5	5
	GenBank link	JAWJLF000000000	JAWJLG000000000	JAWJLH000000000
Annotation	Gene no.	4029	4521	4603
	tRNA no.	83	84	86
	rRNA no.	34	32	41
Sequence typing	MLST	18	18	18
	ERIC	I	I	I

One loopful (10 µL) of bacterial cells from each confirmed isolate was washed with 1 mL of DPBS (Gibco, Life Technologies, Billings, USA) and centrifuged at 14,000 × *g* for 5 minutes. DNA was extracted from the pellet using the QIAamp DNA Mini Kit (Qiagen, Hilden, Germany). DNA fragment length distribution was assessed using the Agilent TapeStation 4200 following the DNA ScreenTape HS D5000 protocol, and DNA concentration was quantified with the Qubit dsDNA HS Assay protocol (Thermo Fisher Scientific, Waltham, MA, USA).

Libraries were prepared for Illumina MiSeq (Illumina, San Diego, CA, USA) and Oxford Nanopore Technologies (ONT) (Oxford, UK) MinION.

For the MiSeq libraries, the Illumina DNAprep and Nextera DNA CD indices were used. Sequencing was performed on a MiSeq platform for 300-nucleotide pair-end sequencing with the MiSeq Reagent v3, 600-cycle chemistry.

Library preparation for ONT MinION was performed using the Rapid gDNA Barcoding SQK-RBK114.24 protocol. A pooled single library was loaded onto a MinION flow cell (R9.4.1). Guppy v6.5.7 was used for base-calling with model dna_r10.3_450bps_hac.

Fastq reads produced by the sequencing instruments were quality controlled using FastQC v0.11.9 (http://www.bioinformatics.babraham.ac.uk/projects/fastqc/) and nanoQC v0.90.4 (3). Low quality and vector/primer reads were excluded using Trimmomatic v0.39 (4).

ONT reads were assembled using Canu v2.2 (5), and Illumina and ONT data were hybrid assembled using Unicycler v0.5.0 (6). Unicycler assemblies were consensus called using Rebaler v0.2.0 (https://github.com/rrwick/Rebaler) with the Canu assembly as reference. Genome completeness was evaluated using BUSCO v5.4.7 (7) and the bacterial database odb_10. MLST sequence type was obtained using Krocus v1.0.3 (8) and the pubMLST database (9) for *Paenibacillus larvae*. ERIC type was derived from MLST as described in reference (10). Taxonomy classification was verified using Type Strain Genome Server (TYGS) (11), showing *Paenibacillus larvae* strain ATCC 9545 as the closest relative. Genomes were annotated at submission time with PGAP v6.6. All programs ran with standard parameters. Table 1 presents read data and assembly stats.

## Data Availability

Genomes and raw reads have been deposited in GenBank and SRA. Links to the public databases are included in Table 1.
